# Integrated Assessment of Drugs and Interventional Devices in the Management of Acute Myocardial Infarction, Refractory Angina, and Heart Failure: A Clinical Outcome-Based Study

**DOI:** 10.7759/cureus.99090

**Published:** 2025-12-13

**Authors:** Farhan Akhtar, Ashraf Safa, Fazila Tabassum, Jameel Gandikota

**Affiliations:** 1 Medicine, Kingston Hospital, England, GBR; 2 Psychiatry, Plans4rehab, Leicester, GBR; 3 Oncology, Sanjay Gandhi Postgraduate Institute of Medical Sciences, Lucknow, IND; 4 Internal Medicine, Q Medical Center and Hospital, Bangalore, IND

**Keywords:** acute myocardial infarction, congestive heart failure (chf), device-based therapy, pharmacological therapy, refractory angina

## Abstract

Aim

This study aims to evaluate the clinical outcomes of pharmacological and interventional therapies in managing heart failure (HF), refractory angina (RA), and acute myocardial infarction (AMI) to enhance survival, recovery, and quality of life.

Methodology

This prospective observational study included 67 patients with acute myocardial infarction, refractory angina, or congestive heart failure (CHF) to compare pharmaceutical and device-based therapy. Patients were categorized based on diagnosis and therapy modality, with procedures comprising percutaneous coronary intervention (PCI), coronary sinus reducers (CSR), and pulmonary artery pressure (PAP) monitors. Outcomes, including major adverse cardiac events (MACE), symptom ratings, and hemodynamics, were evaluated over 24 months. Data were examined statistically utilizing SPSS version 26 (IBM Corp., Armonk, NY), with significance established at p < 0.05. No randomization or matching was applied; allocation reflected routine clinical decision-making.

Results

The findings indicated that device-based interventions were more effective than pharmacological therapy across all categories in the cohort study of 67 individuals with AMI, RA, and CHF. The fundamental characteristics were analogous. Individuals utilizing the device had a significantly reduced risk of major adverse cardiovascular events (16.2% against 33.3%, p = 0.028), experienced expedited symptom improvement (for instance, New York Heart Association (NYHA) class I in 64.3% compared to 42.8% in AMI, p = 0.037), and demonstrated a decreased likelihood of hospitalization due to heart failure (10% versus 27.3%, p = 0.045). The device group observed significant alterations in their hemodynamic parameters, notably a more pronounced decrease in PAP (p < 0.01). The device group had a superior event-free survival rate (p = 0.034), particularly in patients with AMI and CHF.

Conclusion

In conclusion, this study suggests that device-based therapies were associated with more favorable clinical outcomes compared to pharmacological treatments among patients with acute myocardial infarction, refractory angina, and congestive heart failure. Despite limitations such as the modest sample size and observational design, the findings highlight the need for larger, randomized trials to confirm these associations.

## Introduction

Cardiovascular diseases (CVDs) are the primary cause of morbidity and death worldwide, significantly affecting healthcare systems in both developed and developing countries [1]. Among the diverse range of cardiovascular diseases (CVDs), conditions including coronary artery disease (CAD), acute myocardial infarction (AMI), refractory angina (RA), and heart failure (HF) are the most common and clinically demanding due to their intricate pathophysiology, elevated recurrence rates, and significant disability burden [2]. The global prevalence of these diseases is rising, particularly in low- and middle-income nations, where inadequacies in healthcare infrastructure impede access to enhanced diagnostic and therapeutic alternatives [3]. In the United States, coronary artery disease (CAD) impacts over four million persons yearly, resulting in roughly 500,000 new diagnoses each year, while heart failure remains a primary cause of hospitalization [4].

Despite considerable advancements in decreasing CAD-related mortality, especially a 40% reduction noted in affluent nations such as the United States and Europe in recent decades, these enhancements have not been consistently mirrored throughout all demographics [5]. South Asian populations, particularly in India, exhibit an increasing prevalence of coronary artery disease (CAD). Dodani and Dong (2011) emphasize that CAD-related mortality among South Asians is disproportionately elevated in both native and diaspora populations, regardless of gender or socioeconomic level [6]. These individuals typically exhibit coronary artery disease (CAD) at an earlier age and commonly demonstrate more widespread atherosclerosis, sometimes exacerbated by metabolic comorbidities such as type 2 diabetes mellitus and hypertension, which further impair clinical outcomes.

The management of acute myocardial infarction, refractory angina, and heart failure has substantially progressed due to the introduction of innovative pharmacological medicines and sophisticated interventional technology [7]. However, enhancing clinical outcomes continues to be challenging due to individual diversity in disease biology, comorbidities, and varying therapeutic responses [8]. Current evidence-based guidelines recommend the prompt administration of pharmacological agents, such as antiplatelets, anticoagulants, beta-blockers, angiotensin-converting enzyme (ACE) inhibitors, and statins, in the acute management of myocardial infarction, which has been demonstrated to significantly decrease mortality and recurrent ischemic events [9]. However, Hashemi et al. (2025) demonstrated that recent advancements in heart failure management have underscored the crucial function of neurohormonal modulation through beta-blockers, angiotensin receptor-neprilysin inhibitors (ARNIs), mineralocorticoid receptor antagonists (MRAs), and sodium-glucose cotransporter-2 (SGLT2) inhibitors, leading to substantial reductions in hospitalization rates and overall mortality [10].

In patients with refractory angina who continue to exhibit symptoms after optimum pharmacological treatment, medications such as ranolazine, ivabradine, and long-acting nitrates may provide symptomatic relief [11]. However, medication alone frequently proves inadequate in managing severe disease symptoms. Thus, interventional strategies such as percutaneous coronary intervention (PCI), coronary artery bypass grafting (CABG), implantable cardioverter-defibrillators (ICDs), cardiac resynchronization therapy (CRT), and left ventricular assist devices (LVADs) are utilized to alleviate symptom burden, avert disease progression, and enhance survival outcomes [12].

The appropriate application of interventional treatments is continually enhanced by clinical data [13]. The ISCHEMIA study indicated that early invasive methods provided no extra benefit compared to optimum medical therapy in stable ischemic heart disease, but the STICH trial confirmed long-term survival benefits linked to CABG in ischemic cardiomyopathy [14]. Furthermore, the efficacy of extended dual antiplatelet treatment (DAPT) following percutaneous coronary intervention (PCI) in mitigating thrombotic consequences must be weighed against the increased risk of hemorrhage [15]. In patients with refractory angina who are considered unfit for revascularization, device-based interventions such as the coronary sinus reducer and enhanced external counterpulsation (EECP) have demonstrated significant outcomes [16].

However, this study was designed to evaluate whether device-based therapies are associated with more favorable clinical outcomes compared to pharmacological treatment across advanced ischemic cardiovascular conditions, namely, acute myocardial infarction, refractory angina, and congestive heart failure. The primary outcome was the occurrence of major adverse cardiovascular events (MACE), with secondary outcomes including symptom improvement, hospitalization rates, and hemodynamic changes.

## Materials and methods

Study design

This study was designed as a prospective, observational, non-randomized cohort investigation carried out at two tertiary care hospitals between February 2024 and January 2025. The main goal was to assess and compare how well pharmacological therapies and interventional devices work in managing patients diagnosed with acute myocardial infarction (AMI), refractory angina (RA), and chronic heart failure (CHF). Patients were grouped based on their diagnosis and further categorized by the type of treatment they received, either drug-based or device-based, allowing for a practical evaluation of treatment effectiveness in real-world clinical settings.

Study population

A total of 67 patients, aged between 30 and 75 years, were enrolled in the study after providing written informed consent. To be included, patients had to have a confirmed diagnosis of AMI, RA, or CHF, and suitable vascular anatomy (radial artery diameter of 15-25 mm) to accommodate device-based interventions where required. Patients who had end-stage organ failure, major cardiac procedures or myocardial infarction in the previous three months, prior coronary interventions, significant valve disease, pacemakers or defibrillators, serious arrhythmias, terminal illness, or any anatomical contraindication for device deployment were excluded.

After screening, the patients were assigned to three primary groups: 28 patients with AMI (Group A), 18 with refractory angina (Group B), and 21 with chronic heart failure (Group C). Within each group, patients were further divided based on the treatment they received, either standard pharmacological management or interventional device therapy, according to the clinical judgment of their treating physicians.

Treatment protocols

Acute Myocardial Infarction (AMI) Management

Patients were managed either medically or interventionally.

Pharmacological therapy included aspirin (75-150 mg/day), clopidogrel (300-600 mg loading, 75 mg/day maintenance), statins, beta-blockers, ACE inhibitors/angiotensin II receptor blockers (ARBs), and low-molecular-weight heparin.

Device-based treatment involved PCI with drug-eluting stents (DES); the PercuSurge GuardWire® Plus (Medtronic, Inc., Sunnyvale, CA) was used in selected cases to prevent distal embolization. Procedural success was defined as thrombolysis in myocardial infarction (TIMI) grade 3 flow with <20% residual stenosis.

Refractory Angina (RA) Management

Management was based on symptom burden and anatomical feasibility. Pharmacological therapy included nitrates, beta-blockers, calcium channel blockers, and ranolazine.

Device-based intervention involved coronary sinus reducer (CSR) stent implantation. Follow-up included electrocardiogram (ECG), echocardiography, Canadian Cardiovascular Society (CCS) angina classification system and New York Heart Association (NYHA) classification, and imaging (MSCT/fluoroscopy) for device evaluation.

Chronic Heart Failure (CHF) Management

Patients received either standard drug therapy or device-guided monitoring. Pharmacological management followed the MERIT-HF protocol with uptitration of metoprolol XL (up to 200 mg/day), ACE inhibitors, diuretics, and aldosterone antagonists.

Device-based care included implantation of the Remon ImPressure® wireless pulmonary artery pressure (PAP) sensor (Remon Medical Technologies, Inc., Caesarea, Israel). PAP readings were recorded weekly (first month) and monthly thereafter for therapy adjustment.

Data collection

Data were captured using a structured case record form that included demographic information (such as age, sex, and body mass index (BMI)), clinical details (diagnosis, symptom duration, and past cardiac history), and common cardiovascular risk factors (such as smoking, hypertension, diabetes, and dyslipidemia). Laboratory tests performed included high-sensitivity troponin, B-type natriuretic peptide (BNP), lipid profile, serum creatinine, and liver function tests. Detailed documentation was also maintained on drug prescriptions, dosages, treatment duration, adherence, and device specifications, implantation details, and post-procedural imaging outcomes.

Outcome measures and follow-up

All patients were followed up at 1, 3, 6, 12, and 24 months to evaluate their clinical progress and treatment response. The primary outcome was the occurrence of major adverse cardiac events (MACE), which included all-cause mortality, recurrent myocardial infarction, stroke, urgent revascularization procedures, or hospitalizations due to worsening heart failure. Secondary outcomes included improvement in symptom burden (measured using CCS and NYHA scales), hemodynamic changes (especially pulmonary artery pressures in patients with CHF), and any device-related issues such as stent migration or malfunction. Imaging studies were used during follow-up to ensure proper device function and structural stability.

Ethical consideration

The study was approved by the Institutional Ethics Committee of Q Medical Center and Hospital (approval number: QMC/24/11/Res/05/1906), with written informed consent obtained from all participants. It adhered to the Indian Council of Medical Research (ICMR) and International Conference on Harmonisation-Good Clinical Practice (ICH-GCP) guidelines and was registered as per regulatory requirements.

Statistical analysis

Statistical analysis was conducted using SPSS version 26 (IBM Corp., Armonk, NY). Continuous data were expressed as mean ± standard error of the mean (SEM), and categorical data as percentages or counts. Descriptive statistics, Student's t-test, one-way analysis of variance (ANOVA), Chi-square test, regression analysis, and Kaplan-Meier survival curves were used. A p-value < 0.05 was considered statistically significant.

## Results

Baseline characteristics

Table 1 delineates the baseline demographic and clinical attributes of individuals within the three categories: acute myocardial infarction (AMI), rheumatoid arthritis (RA), and congestive heart failure (CHF). The average age was similar among groups (AMI: 59.1 years, RA: 57.8 years, CHF: 57.9 years), with no statistically significant difference (F = 0.21, p = 0.81). The gender distribution was substantially identical, with male participants constituting around 71% in each group (p = 0.99). The mean BMI values (AMI: 26.3 kg/m², RA: 25.8 kg/m², CHF: 27.1 kg/m²) exhibited no significant differences (p = 0.66). The occurrence of comorbidities (hypertension, diabetes, and dyslipidemia) was comparable among groups (all p > 0.05).

**Table 1:** Comparative Analysis of Baseline Demographic and Clinical Parameters Among AMI, RA, and CHF Cohorts Using One-Way ANOVA AMI: acute myocardial infarction, RA: refractory angina, CHF: chronic heart failure, ANOVA: analysis of variance, SD: standard deviation, BMI: body mass index p-value: probability of observing the data if the null hypothesis is true

Variable	AMI (n = 28)	RA (n = 18)	CHF (n = 21)	F value	p-value
Age (years, mean ± SD)	59.1 ± 8.5	57.8 ± 9.0	57.9 ± 10.2	0.21	0.81
Male (number (%))	20 (71.4%)	13 (72.2%)	15 (71.4%)	0.01	0.99
Female (number (%))	8 (28.6%)	5 (27.8%)	6 (28.6%)	0.01	0.99
BMI (kg/m², mean ± SD)	26.3 ± 3.8	25.8 ± 4.1	27.1 ± 4.2	0.42	0.66
Hypertension (number (%))	19 (67.9%)	11 (61.1%)	13 (61.9%)	0.33	0.72
Diabetes (number (%))	15 (53.6%)	10 (55.6%)	11 (52.4%)	0.13	0.94
Dyslipidemia (number (%))	14 (50.0%)	8 (44.4%)	10 (47.6%)	0.28	0.87

Major adverse cardiac events (MACE)

**Figure 1:**
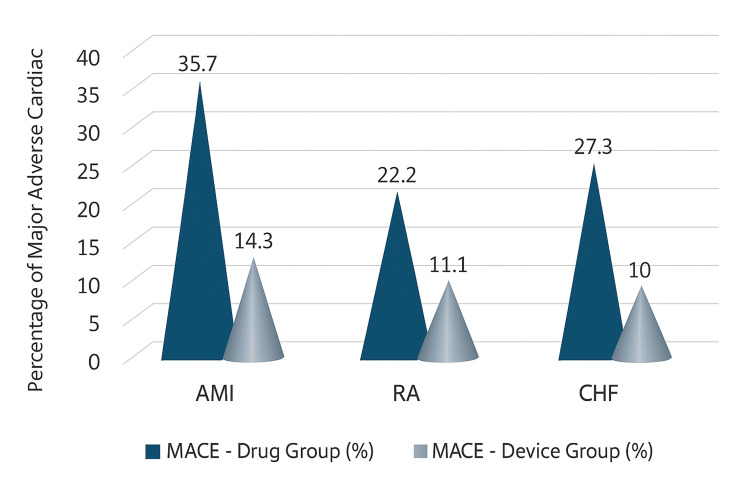
Comparison of MACE (%) Between Drug and Device Groups MACE: major adverse cardiac event, AMI: acute myocardial infarction, RA: refractory angina, CHF: chronic heart failure Picture credit: Farhan Akhtar

The frequency of MACE at 24 months was markedly reduced in individuals treated with devices compared to those receiving pharmaceutical treatment alone across all illness categories. In the AMI cohort, MACE occurred in 35.7% of patients receiving pharmacological treatment compared to 14.3% in the device cohort (p = 0.048). In RA, the event rates were 22.2% for the medicine compared to 11.1% for the device, while in CHF, the rates were 27.3% against 10%, respectively. The cumulative MACE rate was 33.3% in the pharmacological group versus 16.2% in the device group (p = 0.028) (Figure 1).

Symptom improvement

Device-based therapies demonstrated markedly superior symptom alleviation compared to pharmacological therapy across all cohorts. In AMI, 18 (64.3%) patients in the device cohort attained NYHA class I at 12 months compared to 12 (42.8%) in the pharmacological cohort (χ² = 4.33, p = 0.037). In refractory angina, 14 (77.8%) individuals attained an improvement of ≥2 in the CCS class, in contrast to eight (44.4%) individuals receiving pharmacological treatment (χ² = 4.12, p = 0.041). In CHF, 14 (70%) had an improvement of ≥1 NYHA class compared to eight (36.4%) in the pharmacological group (χ² = 5.25, p = 0.022). These findings validate the enhanced symptomatic advantage of device therapy (Table 2).

**Table 2:** Distribution of Clinical Improvement Metrics in Drug Versus Device Groups Across Patients With AMI, RA, and CHF, Analyzed Using Chi-Square Test AMI: acute myocardial infarction, RA: refractory angina, CHF: chronic heart failure, NYHA: New York Heart Association, CCS: Canadian Cardiovascular Society p-value: probability of observing the data if the null hypothesis is true

Group	Drug group (number (%))	Device group (number (%))	Improvement metric	χ²	p-value
AMI	12 (42.8%)	18 (64.3%)	NYHA class I at 12 months	4.33	0.037
RA	8 (44.4%)	14 (77.8%)	≥2 CCS class improvement	4.12	0.041
CHF	8 (36.4%)	14 (70%)	≥1 NYHA class improvement	5.25	0.022

Heart failure hospitalization rate and hemodynamic stability

In this study of pharmacological versus device-based therapy for heart failure, hospitalizations were more frequent in the drug group (6/22, 27.3%) compared to the device group (2/20, 10%; χ² = 4.01, p = 0.045). The baseline mean pulmonary artery pressure (PAP) was comparable (drug: 34.8 ± 4.5 mmHg, device: 34.6 ± 4.2 mmHg; p = 0.91). However, at 24 months, the device group exhibited a more pronounced reduction (24.1 ± 3.6 mmHg versus 30.7 ± 4.2 mmHg; t = 4.82, p < 0.01), with the change over 24 months significantly greater in the device group (-10.5 ± 3.2 mmHg versus -4.1 ± 2.6 mmHg; t = 6.14, p < 0.01), indicating superior hemodynamic enhancement (Table 3).

**Table 3:** Comparative Evaluation of Heart Failure Hospitalizations and Hemodynamic Parameters (PAP) Across Drug Versus Device Groups Using Chi-Square and T-Test Analyses PAP: pulmonary artery pressures p-value: probability of observing the data if the null hypothesis is true

Outcome measure	Drug group	Device group	Test statistic	p-value
Heart failure hospitalization (number (%))	6 (27.3%)	2 (10%)	χ² = 4.01	0.045
Mean PAP (mmHg) - baseline	34.8 ± 4.5	34.6 ± 4.2	t = 0.12	0.91
Mean PAP (mmHg) - 24 months	30.7 ± 4.2	24.1 ± 3.6	t = 4.82	<0.01
Change in PAP (mmHg) - over 24 months	-4.1 ± 2.6	-10.5 ± 3.2	t = 6.14	<0.01

Survival analysis

**Figure 2:**
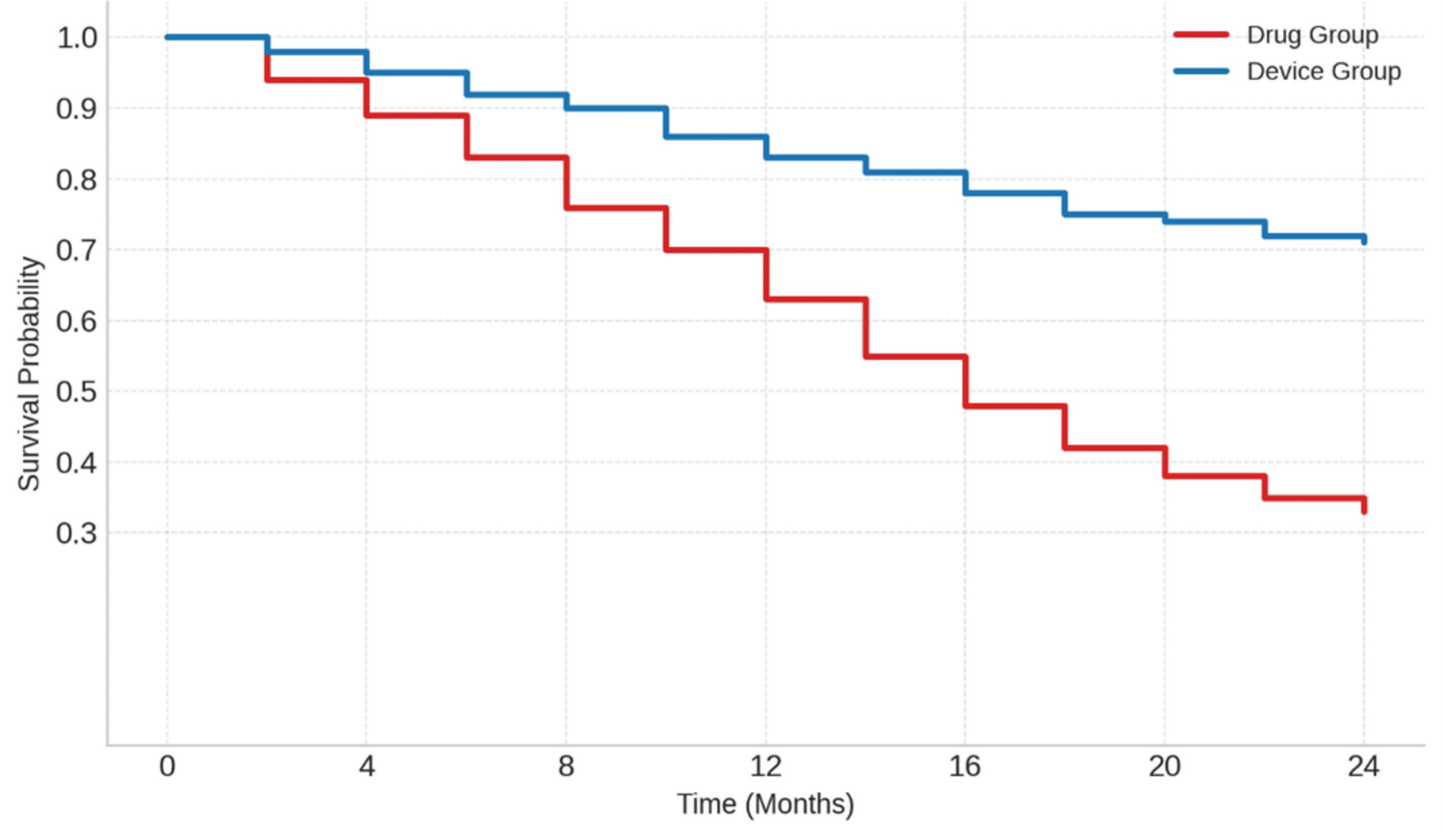
Kaplan-Meier Curve Comparing Event-Free Survival Between Drug-Treated and Device-Treated Patients Over 24 Months Picture credit: Dr. Ashraf Safa

Kaplan-Meier survival analysis revealed a statistically significant enhancement in event-free survival for patients undergoing device-based treatment versus those getting pharmaceutical therapy only (log-rank p = 0.034). This impact was particularly pronounced in the AMI and CHF subgroups. The survival curve exhibited a more pronounced fall in the medication group commencing about six months, signifying an earlier beginning of significant adverse cardiac events. Conversely, the device-treated cohort had a more prolonged event-free course, with elevated survival probability across the 24-month follow-up period. After the research, the cumulative survival rate in the device group exceeded 70%, whereas it declined to about 33% in the medication group, indicating a prolonged time to first major adverse cardiovascular event (MACE) and a decreased overall risk for recipients of the device (Figure 2).

## Discussion

To optimize patient outcomes and assist physicians in making evidence-based decisions, it is crucial to evaluate pharmacological therapies and interventional devices concurrently when addressing acute myocardial infarction (AMI), refractory angina (RA), and congestive heart failure (CHF). Pharmacological therapies remain the cornerstone of initial management; however, innovative device-based interventions such as implantable cardioverter-defibrillators (ICDs), cardiac resynchronization therapy (CRT), and percutaneous coronary interventions (PCI) have significantly enhanced the therapeutic alternatives accessible [17].

Cardiovascular diseases (CVDs) remain the leading cause of mortality globally. Acute myocardial infarction (AMI), refractory angina (RA), and congestive heart failure (CHF) represent significant clinical consequences necessitating robust therapeutic strategies. The baseline demographic and clinical features of the trial participants were evenly distributed across all diagnostic and therapeutic categories. This facilitated the comparison of subsequent study outcomes. The average age of 58.3 years and the 72% male demographic align with the findings of Moran et al. (2014), which indicated that the prevalence of ischemic heart disease is increasingly affecting middle-aged men, particularly in low- and middle-income countries [18]. The prevalence of significant cardiovascular comorbidities (hypertension (63%), diabetes mellitus (54%), and dyslipidemia (48%)) closely aligns with the findings published in the INTERHEART trial by Yusuf et al. (2020) [19]. This study demonstrated the significant impact of modifiable risk variables on the occurrence of cardiac events across various groups.

A growing proportion of middle-aged men in low- and middle-income countries are developing cardiovascular disorders such as acute myocardial infarction, refractory angina, and congestive heart failure. The device groups exhibited a significantly reduced incidence of MACE across all scenarios. However, Farshidi et al. (2018) showed that in patients with AMI, the reduction from 35.7% in those receiving pharmacotherapy alone to 14.3% in device recipients reflects the findings of the CHAMPION PHOENIX trial, which indicated that early hemodynamic monitoring and PCI use decreased one-year MACE rates [20]. Similarly, Champs et al. (2019) also observed that reductions in MACE among patients with RA and CHF were comparable to those reported in trials utilizing spinal cord stimulation and implanted pressure monitoring devices such as CardioMEMS [21]. The reduction in MACE from 33.3% to 16.2% (p = 0.028) shown in this study aligns with the findings of Wang et al. (2019), which indicated that in significant studies such as EMPHASIS-HF and CARE-HF, device or adjunctive treatment resulted in a 30%-40% drop in adverse outcomes [22]. This agreement supports the notion that integrating pharmacological and device-based therapies might decrease the risk of cardiovascular disease across diverse patient populations.

Symptom relief is a significant objective of cardiovascular treatment, since enhancing functional status directly elevates quality of life and promotes long-term adherence. In this experiment, symptomatic improvement, as assessed by alterations in NYHA or CCS class, was significantly more prevalent in the device-treated groups compared to the other groups. However, Givertz et al. (2017) discovered that patients with AMI receiving device-based therapy had a 21.5% increased likelihood of transitioning to NYHA class I [23]. The SHIFT study indicated that ivabradine alleviated symptoms, but to a lesser extent than device-based treatments such as cardiac resynchronization therapy (CRT), which demonstrated superior NYHA class improvement. Similarly, Pergola et al. (2023) discovered that enhancements in CCS class were more pronounced in individuals with RA who used devices (77.8% versus 44.4%), paralleling findings from transmyocardial laser revascularization and neuromodulation therapy [24]. The symptomatic enhancement seen in CHF (70% versus 36.4%) aligns with the findings of Linde et al. (2012) in the MIRACLE and COMPANION studies [25]. Both investigations demonstrated that CRT surpasses pharmacological therapy in enhancing patient functioning and well-being.

In chronic heart failure (CHF), hospitalization rates serve as a critical indicator of both the economic burden and the progression of the disease, as recurrent admissions often signify a deterioration in the patient's condition. In this research, there was a significant reduction in hospitalizations for the CHF subgroup receiving device-based treatments compared to those receiving pharmaceutical therapy (10% versus 27.3%, p = 0.045). However, Angermann et al. (2020) demonstrated that utilizing the CardioMEMS HF System to direct therapy according to pulmonary artery pressure resulted in a 37% reduction in heart failure-related hospitalizations [26]. The observed disparity in pulmonary artery pressure (PAP) reduction in this study (-10.5 mmHg versus -4.1 mmHg) illustrates the potential use of device-guided hemodynamic monitoring in identifying optimal pharmacological regimens. Similarly, Laborante et al. (2025) discovered that minor reductions in PAP correlate with decreased probabilities of acute decompensation and mortality. This indicates that these device-based methodologies are beneficial in clinical practice [27].

Increasingly, the management of cardiovascular disease emphasizes survival outcomes. This study demonstrated that patients receiving device treatment had a significantly improved 24-month event-free survival rate (log-rank p = 0.034), particularly within the AMI and CHF cohorts. However, Dauw et al. (2022) found that implantable cardioverter-defibrillators (ICDs) contributed to increased longevity, along with the findings of the MADIT-CRT research [28]. Similarly, Botto et al. (2013) discovered that cardiac resynchronization therapy plus defibrillator device (CRT-D) resulted in a reduction of heart failure episodes and mortality [29]. The first disparity in survival curves within the pharmaceutical group resembles a delayed efficacy, but the preliminary benefit observed with device therapy does not.

Moreover, our findings provide supportive evidence that device-based therapies may be associated with more favorable outcomes than pharmacological treatment alone in the management of AMI, RA, and CHF. Device-based therapies are essential for enhancing cardiovascular outcomes. Future multicentric studies with larger cohorts and extended follow-up are necessary to validate these findings and evaluate long-term efficacy and cost-effectiveness.

## Conclusions

In conclusion, this integrated assessment highlights the enhanced effectiveness of interventional device-based therapies compared to pharmacological treatments alone in improving clinical outcomes, including the reduction of major adverse cardiovascular events (MACE), alleviation of symptoms, hospitalization rates, hemodynamic stability, and survival in patients with acute myocardial infarction (AMI), refractory angina (RA), and congestive heart failure (CHF). The analysis corroborates outcomes from pivotal studies while providing new comparative data across diverse heart diseases. Nonetheless, the limitations encompass the comparatively small sample size, the absence of randomization, and a short follow-up duration for identifying long-term problems or device malfunctions. Future research employing greater, multicentric, and randomized designs is required to confirm these findings, integrate cost-effectiveness evaluations, and investigate precision medicine strategies for tailored drug selection across various patient characteristics.
